# Ultrafast Tunable Terahertz-to-Visible Light Conversion through Thermal Radiation from Graphene Metamaterials

**DOI:** 10.1021/acs.nanolett.3c00507

**Published:** 2023-04-28

**Authors:** Igor Ilyakov, Alexey Ponomaryov, David Saleta Reig, Conor Murphy, Jake Dudley Mehew, Thales V.A.G. de Oliveira, Gulloo Lal Prajapati, Atiqa Arshad, Jan-Christoph Deinert, Monica Felicia Craciun, Saverio Russo, Sergey Kovalev, Klaas-Jan Tielrooij

**Affiliations:** † Institute of Radiation Physics, Helmholtz-Zentrum Dresden-Rossendorf, Bautzner Landstr. 400, 01328 Dresden, Germany; ‡ Catalan Institute of Nanoscience and Nanotechnology (ICN2), BIST and CSIC, Campus UAB, Bellaterra, Barcelona 08193, Spain; § Centre for Graphene Science, University of Exeter, Exeter, EX4 4QF, U.K.; ∥ Department of Applied Physics, TU Eindhoven, Den Dolech 2, 5612 AZ, Eindhoven, The Netherlands

**Keywords:** terahertz radiation, frequency conversion, ultrafast thermal emission, graphene, electrical gating, metamaterial

## Abstract

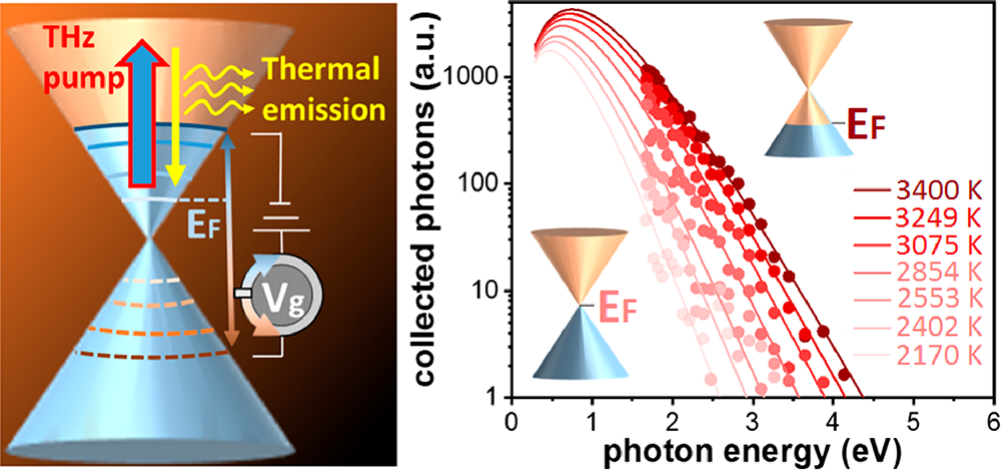

Several technologies, including photodetection, imaging, and data communication, could greatly benefit from the availability of fast and controllable conversion of terahertz (THz) light to visible light. Here, we demonstrate that the exceptional properties and dynamics of electronic heat in graphene allow for a THz-to-visible conversion, which is switchable at a sub-nanosecond time scale. We show a tunable on/off ratio of more than 30 for the emitted visible light, achieved through electrical gating using a gate voltage on the order of 1 V. We also demonstrate that a grating-graphene metamaterial leads to an increase in THz-induced emitted power in the visible range by 2 orders of magnitude. The experimental results are in agreement with a thermodynamic model that describes blackbody radiation from the electron system heated through intraband Drude absorption of THz light. These results provide a promising route toward novel functionalities of optoelectronic technologies in the THz regime.

The current and future needs of society for high-frequency communication, imaging, and sensing have prompted great interest in THz photonics and optoelectronics research and development.^1,2^ Particularly interesting applications exist in fields ranging from homeland security to quality inspection and from astronomy to wireless communication. The THz range is however a technologically less developed part of the electromagnetic spectrum, due to its location between the realms of electronics and optics.

The conversion of signals between different frequencies provides a powerful approach to exploit the advantageous properties of specific frequency regions. For example, in the method of electro-optical sampling a THz field is encoded onto the polarization of near-infrared light, which is then efficiently measured with conventional Si detectors.^3^ Another example is imaging applications, where recent studies showed efficient photodetection by upconverting broad-band THz light to near-infrared light using a cryogenic quantum well device^4^ and graphene.^5^ Frequency conversion is of particular importance in communication technologies, where optical interconnects provide the crucial link between electronic signals that are processed in the devices of end users and optical signals that are transported through optical fibers that provide a higher information transmission capacity than electronic channels. Similar transducers will likely be required for 6G wireless communication systems, where (sub-)THz carrier frequencies will be deployed. An efficient and tunable mechanism of converting THz waves to the visible or near-infrared domain is thus highly desirable, yet currently elusive.

Gapless 2D materials with massless charge carriers, such as graphene and topological insulators, are highly promising for THz optoelectronics due to the combination of high carrier mobility and low electronic heat capacity of the 2D massless electron gas. Indeed, graphene has been studied and exploited quite extensively in the THz regime. For example, it has been used for efficient THz detection,^6−8^ active ultrafast modulation,^9,10^ and THz harmonic generation.^11−14^ Two-dimensional materials furthermore offer the possibility to control their properties through electrostatic gating. This has, for example, enabled electronic control over frequency conversion through harmonic generation in graphene and semiconducting layered materials^13,15−19^ and electrical control of the photoluminescence of graphene excited by near-infrared light.^20^

Graphene is therefore an interesting candidate material system for tunable THz-to-visible frequency conversion. Several studies have shown that graphene can emit broad-band light in the visible range upon excitation by ultrashort pulses, typically in the near-infrared range. This effect was ascribed to radiative recombination,^21^ thermal emission,^20,22,23^ and collisions between electrons and holes.^24^ Graphene emission in the visible range has also been observed upon excitation by intense single-cycle THz pulses,^25^ where the observed broad-band emission was attributed to Landau–Zener interband transitions during THz excitation. A clear understanding and experimental validation of the mechanism behind THz-induced visible emission from graphene is however currently lacking.

In this work we provide a demonstration of ultrafast, gate-tunable conversion of THz light to UV–visible light and find that our results are in agreement with a thermal radiation mechanism from the heated electron system of graphene. This mechanism works as follows: incident THz radiation is absorbed by the mobile charge carriers in graphene via Drude absorption. The carriers in the electronic system rapidly exchange energy, resulting in a distribution with an elevated electron temperature. This leads to thermal radiation in the visible and near-infrared for temperatures in the range of a few thousand Kelvin. We study three different graphene samples (see Supporting Information Note 1 for details). Sample A (see Figure 1a) consists of single-layer graphene grown by chemical vapor deposition (CVD) on a 1 mm thick quartz glass substrate. The graphene layer is in contact with two metal electrodes that act as source and drain, allowing for measurement of the resistance. Two gate electrodes at opposite lateral edges of the graphene sheet are in contact with the polymer electrolyte LiClO_4_:poly(ethylene oxide), but not with the graphene sheet, thus allowing for electrostatic gating. By applying a gate voltage on the order of 1 V, we can shift the Fermi energy *E*_F_ up to around 0.4 eV.^13^ Sample B consists of a few-layer graphene film grown by CVD and intercalated with ferric chloride (FeCl_3_) (see Supporting Information Note 1).^26^ Hole doping from FeCl_3_ increases the carrier density in the graphene layers, while leaving this material highly transparent to visible and near-infrared light.^27^ We estimate the Fermi energy in Sample B to be 0.55 eV (see Supporting Information Figure S1). Finally, Sample C is a grating-graphene metamaterial sample, where CVD graphene is covered by metallic strips with a metal width of 18 μm, separated by gaps with a width of 2 μm. This implies that 90% of the graphene area is covered by metal, and there is 10% active graphene area. This Sample C leads to increased light–matter interaction inside the gap, where the field enhancement factor is approximately 5, and to an overall increased absorption by a factor of 2.5.^12^

**Figure 1:**
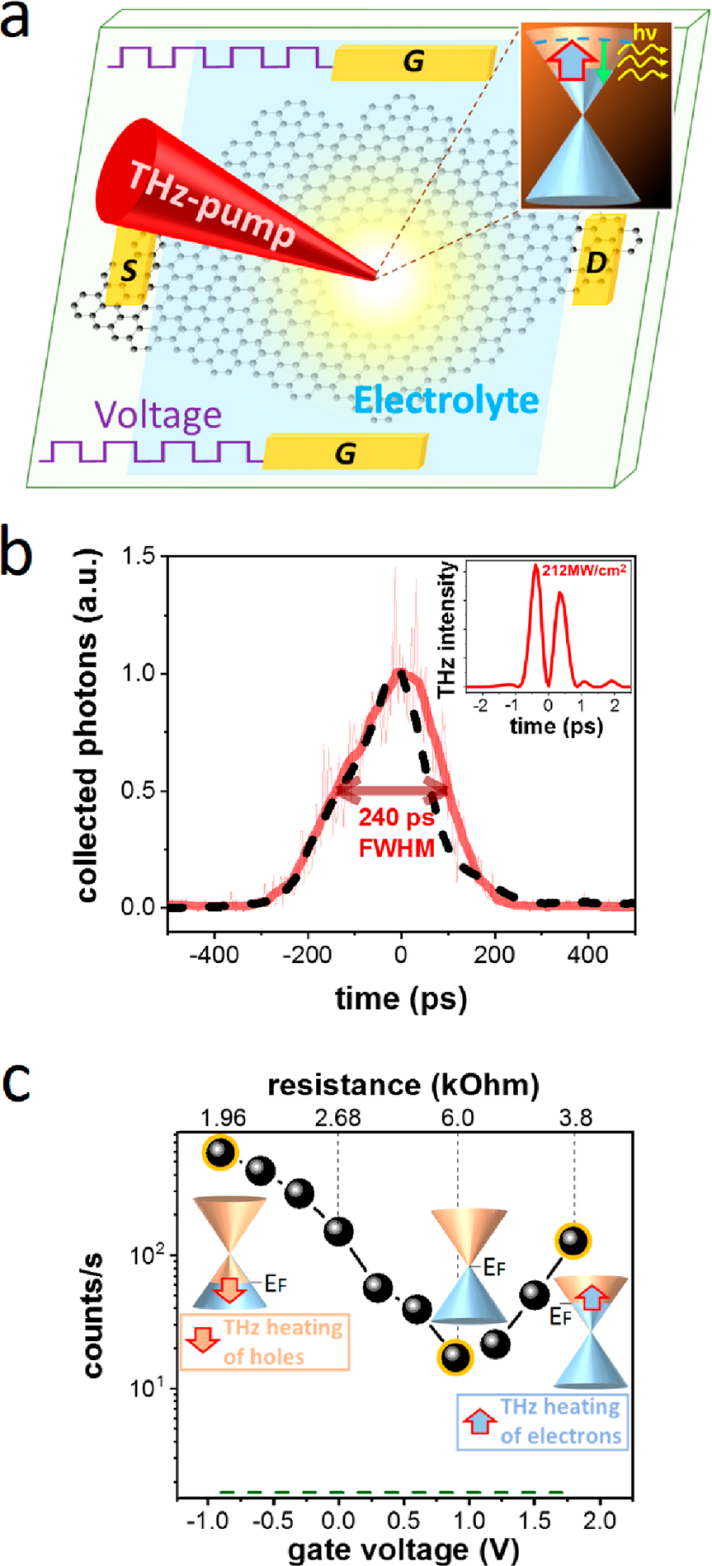
(a) Schematic of the THz-induced photoemission generation from an electrically gated graphene sample (Sample A). Drain (D) and source (S) electrodes are in contact with the graphene layer. An electrolyte gate covers the graphene layer and gate (G) electrodes. Applying an electrical voltage to the electrolyte shifts the Fermi energy in graphene. (b) Temporal dependence of the observed emission (thin red line represents individual data points; thick red line represents the smoothed values) measured by time-correlated single photon counting. The detected fwhm is very close to the instrument response function of 210 ps (black dashed line), determined by using incident laser pulses at 800 nm with a duration of 30 fs. Inset: electro-optically detected time profile of the THz pump-pulse intensity. (c) We control the THz-induced emission in the visible range using the gate voltage, where the emission magnitude (in counts/s) changes by more than 30 times. We simultaneously measured the graphene resistance by applying a small bias voltage between S and D electrodes and the measuring current (see top horizontal axis). The highest resistance occurs at the Dirac point for *V*_D_ = 0.9 V, where THz absorption is reduced, resulting in a lower electron temperature and therefore weaker emission in the visible region. The green-dashed horizontal line is a background level detected signal when the substrate with electrolyte and without graphene is used.

In our experiments, we excite these samples using single-cycle THz pulses centered around 0.5 THz and collect emitted photons over an angle of approximately 10° (see Supporting Information Note 2 for additional information about the experiments). The peak THz fluence is on the order of 176 μJ cm^–2^, which is incident on the graphene samples at an angle of 45°. We first detect the optical emission using time-correlated single-photon counting (TCSPC) and Sample A. We observe visible emission with a duration of around 240 ps (full-width at half-maximum, fwhm), which is similar to the instrument response function (see Figure 1b). This shows that THz-induced visible emission is only present during a very short time scale after interaction with a THz pulse, which is interesting, because it allows for THz-to-visible light conversion at high modulation frequencies.

We next demonstrate electrostatic control over visible light emission upon excitation by THz pulses (see Figure 1c). We observe an on/off ratio of more than 30 for the visible emission from Sample A, while the electrical resistance of the graphene layer changes by around a factor of 3. The number of emitted photons increases upon increasing the doping level. This dependence is qualitatively different from that observed for visible emission induced by ultrashort near-infrared pulses instead of THz pulses. In that case, the emission gradually decreases upon increasing the Fermi energy, followed by a steep decrease when the Fermi energy exceeds half of the photon energy of the excitation pulse.^20^ In both cases, we understand the trends intuitively from the Fermi-level-dependent light absorption. For near-infrared excitation, the steep decrease in the emission is due to Pauli blocking, which strongly limits interband absorption for elevated Fermi energy. For THz excitation, absorption occurs due to the intraband Drude response. Upon increasing the doping level the number of free carriers increases, which leads to increased THz absorption.

In order to study these qualitative trends more quantitatively, we examine the emission spectra as a function of Fermi energy. In Figure 2a, we show the experimentally obtained emission spectra using gate-tunable Sample A. The THz-induced emission spectra are very broad and extend beyond the experimentally observed range (1.5–4 eV). We describe these spectra by blackbody radiation using *P*_em_(ν)= C·(*h*ν)^*2*^/[1 + exp(*h*ν/(2·*T*_e_)]^2^, where *P*_*em*_ is the emitted power, ν is the emitted photon frequency, *h* is Planck’s constant, *T*_e_ is the graphene sheet electronic temperature, and *C* is a constant that captures the emission and collection efficiencies of the experimental setup. Importantly, all experimental spectra can be described by thermal emission with a fixed amplitude *C* and a temperature *T*_*e*_ that depends on the Fermi energy. Figure 2b shows the obtained temperatures.

**Figure 2:**
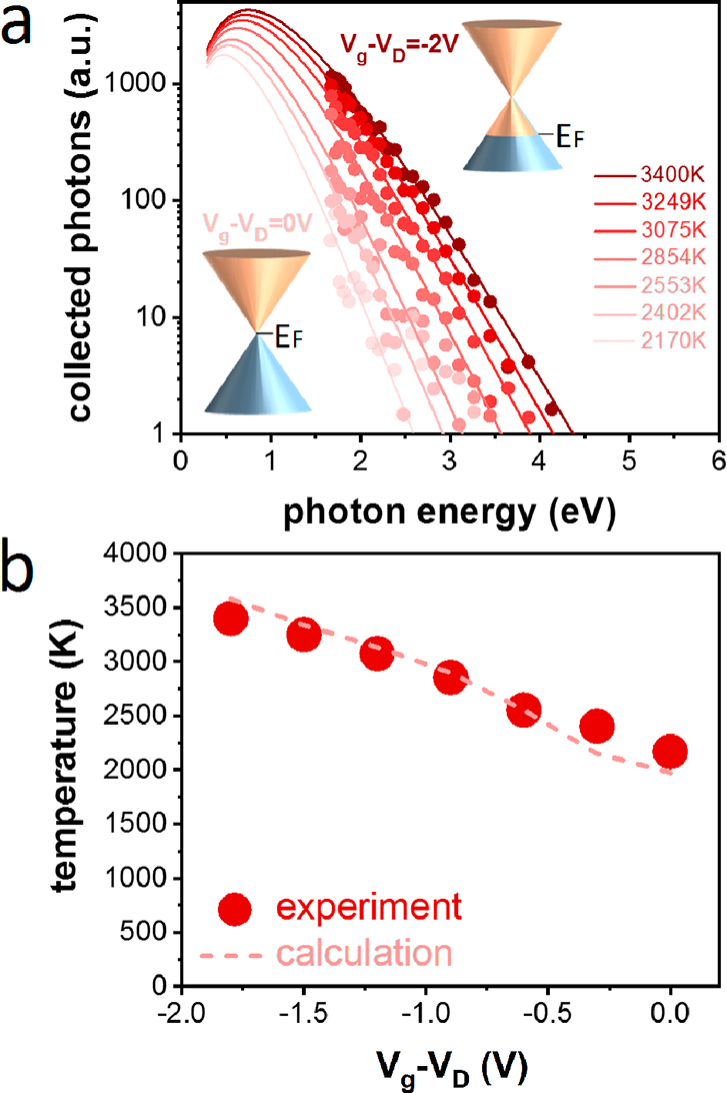
THz-induced emission spectra and corresponding radiation temperatures. (a) Experimental photoemission spectra at different gate voltages (dots) together with blackbody radiation spectra (lines), where the electron temperature is the only freely adjustable parameter, while the amplitude scaling factor is kept fixed for all measurements. The optical spectra are normalized by the response curve of the spectrometer. (b) The electron temperatures obtained from the blackbody emission spectra (dots) and from a thermodynamic model of THz-induced electron heating (dashed line; see Supporting Information Note 3), as a function of the gate voltage (*V*_D_ = 0.9 V).

We test the validity of the thermal emission mechanism by comparing the obtained temperatures with a calculation of the electron temperature of graphene upon THz light absorption. In this model (see Supporting Information Note 3), all energy that is absorbed from THz radiation is directly converted into electronic heat. The temperature that is reached is governed by the electronic heat capacity, where we use the regime *T*_e_ > *T*_F_ = *E*_F_/*k*_B_, with *T*_F_ being the Fermi temperature and *k*_B_ the Boltzmann constant.^28^ In this regime, the heat capacity does not depend on the Fermi energy and the only parameter that changes with *E*_F_ is the THz absorption, which depends on the sheet conductivity σ. Since we simultaneously perform gate-dependent electrical conductivity measurements through the source-drain contacts of our sample (see Figure 1b), we obtain the Fermi-energy-dependent absorption from the sheet conductivity using the thin-film approximation (see Supporting Information Note 3). The resulting electron temperatures from this simple thermodynamic model agree well with the experimental data, which adds credibility to the assignment of the THz-induced emission to an electronic heat effect.

Since we observe that increasing the Fermi energy leads to stronger THz-induced visible emission, we study if we can further increase the emission by using a highly doped FeCl_3_ intercalated few-layer graphene sample (sample B). We also study if we can increase the emission using a grating-graphene metamaterial with enhanced light–matter interaction. We prepared Sample C, which consists of a graphene sheet covered by metallic stripes, as described in ref (12). Figure 3a shows schematics of Samples B and C. For these measurements, we vary the incident THz power and measure the collected number of emitted photons, as shown in Figure 3b. We observe a monotonous increase, where the optical emission scales roughly quadratically with incident THz power. The observed emission from Samples B and C is stronger than the emission from the gate-tunable sample (Sample A, taken at *V*_g_ – *V*_D_ = −1.8 V), which we ascribe to the higher Fermi energy (Sample B) and to an enhanced light–matter interaction (Sample C). We note that the Fermi energy in Sample B is 0.55 eV, which could affect interband transitions below ∼1.1 eV through Pauli blocking. Since we examine spectra well above 1.5 eV, we are not sensitive to these effects. At the highest THz power, we find that the grating-graphene metamaterial emits up to 2 orders of magnitude more photons than the gate-tunable graphene.

**Figure 3:**
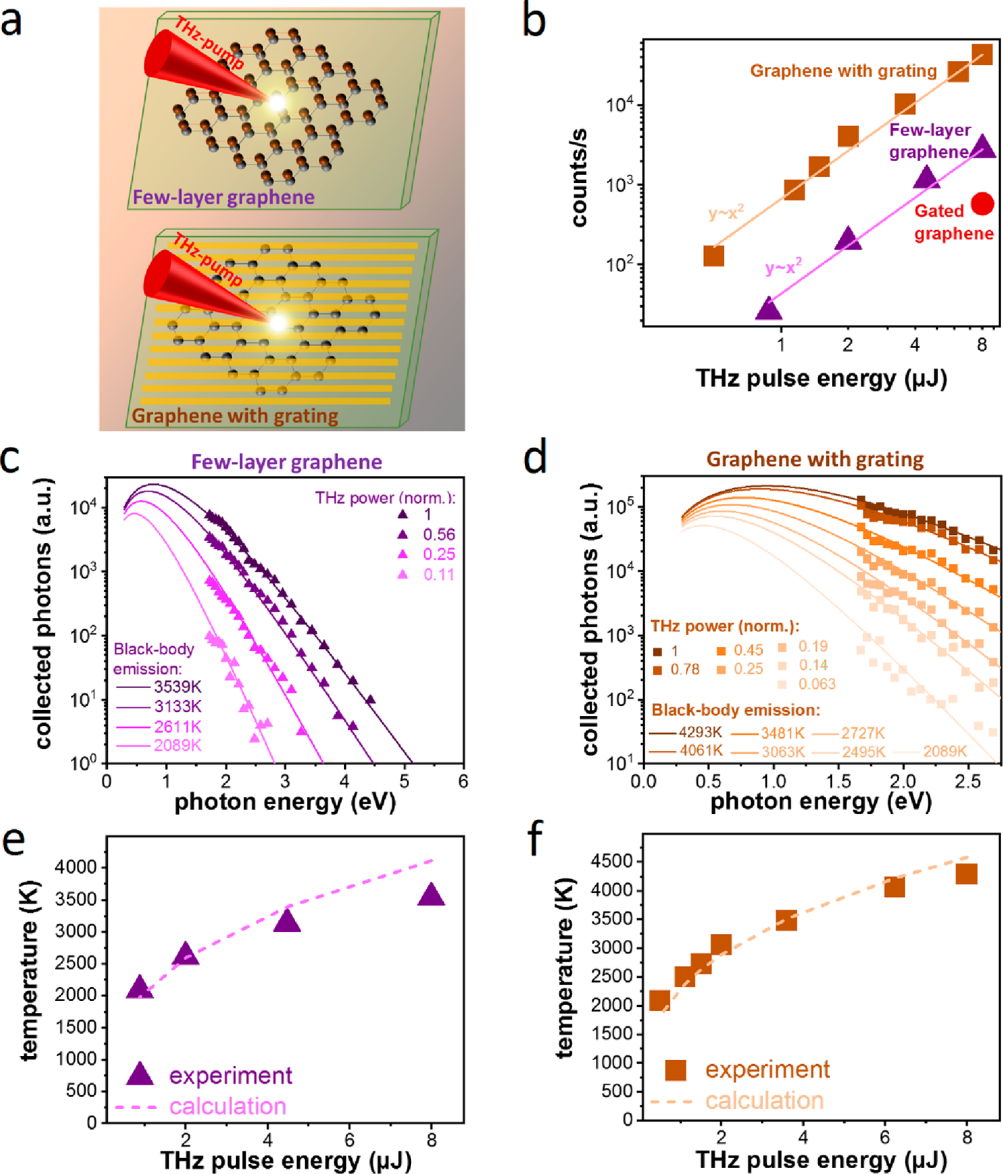
Increasing the THz-induced visible emission. (a) Schematic of the THz-induced photoemission generation from an intercalated few-layer graphene sample (Sample B) and a grating-graphene metamaterial sample (Sample C). (b) THz-induced visible emission in different samples for increasing incident THz pulse energy. The gated graphene data point is taken at *V*_*g*_ – *V*_*D*_ = −1.8 V. (c, d) Emission spectra and corresponding blackbody radiation temperatures for Samples B (c) and C (d) as a function of the incident THz pulse energy. The temperature is the only freely adjustable parameter for the curves corresponding to each sample. The optical spectra are normalized by the response curve of the spectrometer. (e, f) The electron temperatures obtained from the blackbody emission spectra (dots) and from a thermodynamic model of THz-induced electron heating (dashed line; see Supporting Information Note 3), as a function of THz pulse energy for Sample B (e) and Sample C (f).

In order to understand these results quantitatively, we again describe the experimentally obtained spectra using blackbody radiation with a fixed amplitude *C* and variable temperature *T*_e_ (see Figures 3c,d). We note that the spectra in Figure 3d are taken over a narrower photon energy range because of emission from the metal strips at higher photon energies (see Supporting Information Figure S2). Figure 3e,f shows the obtained temperatures from the blackbody radiation spectra, together with the results from our thermodynamic model. Again, we use the limit *T*_e_ > *T*_F_ and fix *E*_F_ to the value obtained from independent measurements on each of the samples (see Supporting Information Note 3). This means that the only parameter that varies is the absorbed power. For Sample C, we use a grating-induced absorption enhancement of 2.5, based on the simulations in ref (10). The observed agreement again adds credibility to the assignment of the THz-induced emission as a consequence of THz-induced electronic heating.

We will now briefly discuss the results in terms of the mechanism that is responsible for THz-induced visible light emission, followed by a discussion of the speed and efficiency of this conversion mechanism, as well as possible technologies that could be enabled by this mechanism. The experimental spectra of all three samples can be described accurately by blackbody radiation spectra, for a range of incident powers and a range of Fermi energies, and the obtained temperatures are in agreement with those from a simple thermodynamic model. This leads to the conclusion that the observed emission is likely the straightforward result of THz-induced thermal emission from the heated electron system. This is in agreement with earlier observations of efficient THz-induced electronic heating in graphene^29^ and thermal emission of the electron system of graphene upon excitation by ultrashort pulses in the near-infrared.^20,22,23^

The speed of this THz-to-visible conversion mechanism is promising for data communication applications that require GHz, or even THz, modulation frequencies. We observe a temporal response with a subnanosecond time scale, which is an upper limit because it is limited by the temporal response function of our detection system. Indeed, we expect that emission occurs within a ps time window—the time it takes for THz heated electrons to dissipate their energy to phonons.^30^ Thus, this mechanism could allow for ultrahigh-speed modulation of THz-to-optical conversion with a bandwidth up to hundreds of gigahertz, which is interesting for information and communication technologies. We note that high-speed switching can be achieved with a back-gate or top-gate^31^ or by hybridizing electrolyte top-gating with back-gating.^32^ We note that the conversion benefits from an elevated electron temperature, which benefits from the use of large peak powers. For THz pulses that are longer than the cooling time, the efficiency decreases for a given average THz power.

Sample C has the highest optical emission, which corresponds to a 10^–10^ THz-to-visible energy ratio. Since the collection angle in our experiment is roughly 1/400 of the whole sphere, the actual conversion efficiency is significantly higher—approximately 10^–8^. Importantly, the power dependence of the emission scales quadratically with THz power without any signatures of saturation. Thus, we expect to achieve higher THz-to-visible conversion efficiencies under higher incident THz fields. Furthermore, by using metamaterial structures with larger field and absorption enhancement,^33−36^ we expect to reach efficient conversion even for lower incident THz powers, well below the μW range. We note that the absence of saturation is in contrast to recent studies of THz harmonic generation using grating-graphene metamaterials,^12,37^ which we understand as follows. To increase incident power, electronic cooling via phonon emission slows down as shown in ref (30), which limits harmonic generation, as this relies on the ultrafast heating–cooling dynamics.^37^ In contrast, we expect that THz-induced visible emission benefits from slower electronic cooling via phonons, as this currently acts as an efficient competing pathway for thermal emission. Interestingly, suspended graphene demonstrates a 1000-fold enhancement in thermal radiation efficiency compared with substrate-supported graphene, when both are excited with DC electrical current,^38−40^ which could also be a promising approach to enhance the THz-to-visible conversion efficiency. Finally, we point out that we could use nanophotonic structures in order to enhance and control the coupling efficiency of hot carriers to far-field radiation, through plasmon–electron and plasmon–phonon interactions.^41^ Such an approach can also lead to more narrow-band radiation, as shown for mid-infrared emission from graphene coated with nanodisks and excited by ultrashort near-infrared light pulses.^42^

Regarding potential technologies based on this ultrafast and controllable THz-to-visible conversion mechanism, there are several opportunities related to information and communication technologies. For example, since sixth generation (6G) wireless communication systems will likely employ frequency bands above 100 GHz, it could be interesting to have a way to convert signals from telecom photons to THz photons and vice versa. Interestingly, the peak of the THz-induced thermal emission spectra that we obtained occurs around 1 eV, which is close to the most important telecom bands (1550 nm = 0.8 eV). These spectra are determined by the charge carrier temperature, which can be controlled either by THz intensity or by electrical gating. Thus, this mechanism could be a promising avenue toward THz-to-telecom interconnects. The observed effect can be also used for spatiotemporal detection of THz wavepackets, as the THz-induced light emission occurs on a picosecond time scale and the emitted radiation scales linearly with instantaneous THz intensity without any signatures of saturation or threshold. Our findings thus pave the way for a range of THz photonic technologies that require control of THz-to-visible energy conversion at exceptionally high speeds.

## Supporting Information Available

Experimental methods, THz intensity estimation, sample preparation, theoretical calculations of electron temperature, characterization of Sample B, and optical emission from a metamaterial grating sample without graphene (PDF)

## Abbreviations

THz: terahertz
CVD: chemical vapor deposition
TCSPC: time-correlated single-photon counting
fwhm: full-width at half-maximum
6G: sixth generation
