# Supplementary material for: Ultrafast Tunable Terahertz-to-Visible Light Conversion through Thermal Radiation from Graphene Metamaterials
Source: Nano Lett. 2023 Apr 28;23(9):3872–8. doi: 10.1021/acs.nanolett.3c00507 (PMC10176577; doi:10.1021/acs.nanolett.3c00507)
Supplement: Supplementary file 1 — nl3c00507_si_001.pdf [file nl3c00507_si_001.pdf]

Supporting Information: Ultrafast Tunable Terahertz-to-Visible Light Conversion through Thermal Radiation from Graphene Metamaterials

Igor Ilyakov[1,], Alexey Ponomaryov[1], David Saleta Reig[2], Conor Murphy[3], Jake Dudley Mehew[2], Thales V.A.G. de Oliveira[1], Gulloo Lal Prajapati[1], Atiqa Arshad[1], Jan-Christoph Deinert[1], Monica Felicia Craciun[3], Saverio Russo[3], Sergey Kovalev[1,*], Klaas-Jan Tielrooij[2,4,*].*

[1]Institute of Radiation Physics, Helmholtz-Zentrum Dresden-Rossendorf, Bautzner Landstr. 400, 01328 Dresden, Germany

[2]Catalan Institute of Nanoscience and Nanotechnology (ICN2), BIST and CSIC, Campus UAB, Bellaterra, Barcelona 08193, Spain

[3]Centre for Graphene Science, University of Exeter, Exeter, EX4 4QF, UK

[4]Department of Applied Physics, TU Eindhoven, Den Dolech 2, 5612 AZ, Eindhoven, The Netherlands

*i.ilyakov@hzdr.de, s.kovalev@hzdr.de, klaas.tielrooij@icn2.cat.

Supplementary Note 1, Sample Preparation:

The gate-controlled graphene sample (Sample A) contains a monolayer graphene sheet with an area of approximately 1x1 cm^2, grown by chemical vapor deposition (CVD), which was transferred onto an Infrasil quartz substrate. We used optical lithography and evaporation of titanium and gold in order to define source (S) and drain (D) electrodes that are in contact with the graphene sheet, and gate electrodes (G) that are not touching the graphene. The electrodes are wire-bonded to metallic leads mounted on a sample holder for electrical control. The final fabrication step consisted of applying a transparent polymer electrolyte top gate, consisting of PEO and $LiClO_4$ with 8:1 weight ratio in a solution of methanol, that was uniformly deposited on top of the graphene and the two gate electrodes. The graphene resistance was measured using the S and D electrodes, while the Fermi energy was controlled by changing the voltage on the G electrodes. Sample B is a highly doped intercalated few-layer graphene sample, dubbed "graphexeter", which we obtain following references **[S1, S2]**. Briefly, we transferred a few-layer graphene film, grown by CVD on nickel, onto an Infrasil quartz substrate followed by intercalation with ferric chloride ($FeCl_3$). The intercalation

process took place using the vapor transport method in a two-zone furnace. We placed anhydrous $FeCl_3$ powder and the graphene sample at different positions inside a glass tube and evacuated the tube down to a pressure of 10^{-6} mbar to reduce contamination by water molecules. The sample and the $FeCl_3$ were heated for 12 hours at $360°C$ and $315°C$ respectively, which allows for the sublimation of the $FeCl_3$ molecules and their intercalation into the few-layer graphene film. Sample C, the metamaterial-grating graphene sample, was also produced by transferring CVD-grown monolayer graphene (approximately 1x1 cm^2 area) onto an Infrasil quartz substrate. We then applied atomic layer deposition of 2 nm of Al_2O_3 and then used optical lithography and thermal evaporation of 50 nm of gold with titanium in order to create the metallic stripes. These have a width on the order of 20 microns and are separated by a gap of ~2 microns.

Supplementary Note 2, Experimental:

THz pulses were generated in a $LiNbO_3$ crystal by tilted pulse-front technique **[S3]** using Ti:sapphire laser pulses (800 nm, 30 fs, 8 mJ, 1 kHz repetition rate). The focused THz pulses (up to 8 μJ pulse energy) obliquely incident (45 degrees incident angle, TE polarization) onto graphene samples reaching approximately 200 MW/cm^2 peak intensity in the sample position. The THz electric field time trace was measured by free-space electro-optic sampling in 2 mm thick (110) ZnTe. The THz beam waist (approximately 2 mm FWHM) was detected by a pyroelectric array camera at normal incidence. Knowing the THz beam diameter, pulse energy and electric field time-trace the THz intensity was determined (Supplementary Note 4). THz power was varied using two subsequent THz free-space wire-grid polarizers (the second one determines the transmitted THz polarization and its orientation was fixed). The THz-induced PL was collected by a lens in the specular reflection direction within 1 inch aperture at 5 inch distance from the sample and then detected using a time-correlated single photon counter. The spectral information was obtained using an additional built-in monochromator.

Supplementary Note 3, Calculations of electron temperature:

In order to compare the electron temperatures that we obtained from describing the emission spectra by black-body radiation, we also calculated the expected electron temperatures based on the incident power, THz absorption and heat capacity of the graphene sheet using the following procedure. We used an incident THz fluence of F_{in} = 13.5 μJ/cm^2 and a THz absorption A determined by the sheet conductivity σ via the thin film approximation: $A = 1 - \left(1 + \frac{\sigma Z_0}{1+n_s}\right)^{-1}$. Here A is absorption, Z_0 is the free-space impedance, and n_s is the refractive index of the substrate (1.5 for our quartz samples). For gate-controlled graphene sample A, we

used the gate-dependent resistance (R) measurements (see Fig. 1c in the main text) and subtracted an estimated contact resistance of $R_c \sim 1.3$ kΩ, in order to obtain the sheet conductivity $= \frac{1}{R - R_c}$. For samples B and C, we obtained the sheet conductivity using $\sigma = ne\mu$, where n is the carrier density and μ is the charge mobility. For the intercalated few-layer graphene Sample B, we used $n = 2.2 \cdot 10^{13}/\text{cm}^2$ and $\mu = 700\ \text{cm}^2/\text{Vs}$ (from Raman measurements, see Supplement). For grating-graphene metamaterial sample C, we used $n = 4 \cdot 10^{12}/\text{cm}^2$ and $\mu = 2000\ \text{cm}^2/\text{Vs}$ (based on Deinert et al, [**S4**]). We then calculated the absorbed THz fluence as $F_{\text{abs}} = A \cdot F_{\text{in}}$ for Samples A and B, while for Sample C we multiplied this by the absorption enhancement factor of 2.5. Finally, we calculated the electron temperature using: $T_e = \sqrt[3]{T_0^3 + \frac{3F_{\text{abs}}}{\alpha}}$, where T_0 is the initial temperature (300 K) and $= \frac{21.636 \cdot k_B^3}{\pi(\hbar v_F)^2}$, which is valid in the regime $T_e > T_F = E_F/k_B$ [**S5**]. For the simulations we use an incident fluence of 13.5 µJ/cm^2, which is lower than the experimentally estimated fluence. This means that the actual spot size might be a bit larger, for example due to the 45 degree incidence. It could also mean that not all incident power is converted perfectly into electronic heat. Importantly, this lower fluence does not affect any of the functional dependences that we studied, namely how the electron temperature scales with Fermi energy (via the gate voltage) and on incident power.

Supplementary Note 4, THz fluence and intensity:

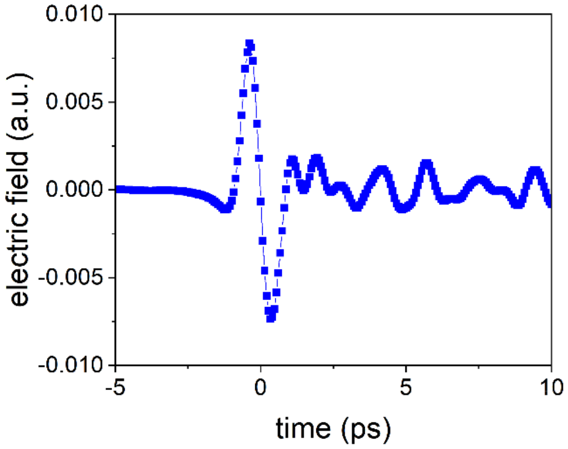

Terahertz electric field distribution.

The THz pulse energy (W) and focal beam waist at full width half maximum ($2 \cdot a$) are 8µJ and 2mm, respectively. To calculate the fluence (F) and intensity (I) we accounted the Gaussian spatial distribution of the THz beam and measured THz electric field time-domain distribution by means of electrooptic sampling. The pulse energy can be described as

$$W = \int_0^\infty F_0 \cdot e^{-(r/a)^2 \cdot ln2} \cdot 2\pi r \cdot dr = \frac{\pi a^2}{ln2} \cdot F_0,$$

where F_0 is a fluence value in the center of the THz beam:

$$F_0 = W \cdot \frac{ln2}{\pi a^2} \approx 176\ \mu J \cdot cm^{-2}$$

Knowing the THz fluence and electric field distribution (E_i) we can estimate the THz intensity (I_i) in absolute units:

$$F_0 = \sum_{i=0}^{N} I_i \cdot \Delta t = I_{max} \cdot \sum_{i=0}^{N} \frac{E_i^2}{E_{max}^2} \cdot \Delta t,$$

Where i is a point number on the time trace, N – the total number of points, I_{max} and E_{max} are the maximal intensity and electric field values and Δt is a time-step value. Then, for I_{max} we get the following value:

$$I_{max} = F_0 \cdot E_{max}^2 \Big/ \left(\sum_{i=0}^{N} E_i^2 \cdot \Delta t \right) = \frac{W \cdot ln2 \cdot E_{max}^2}{\pi a^2 \cdot \sum_{i=0}^{N} E_i^2 \cdot \Delta t} \approx 212\ MW \cdot cm^{-2}.$$

Supplementary Figures:

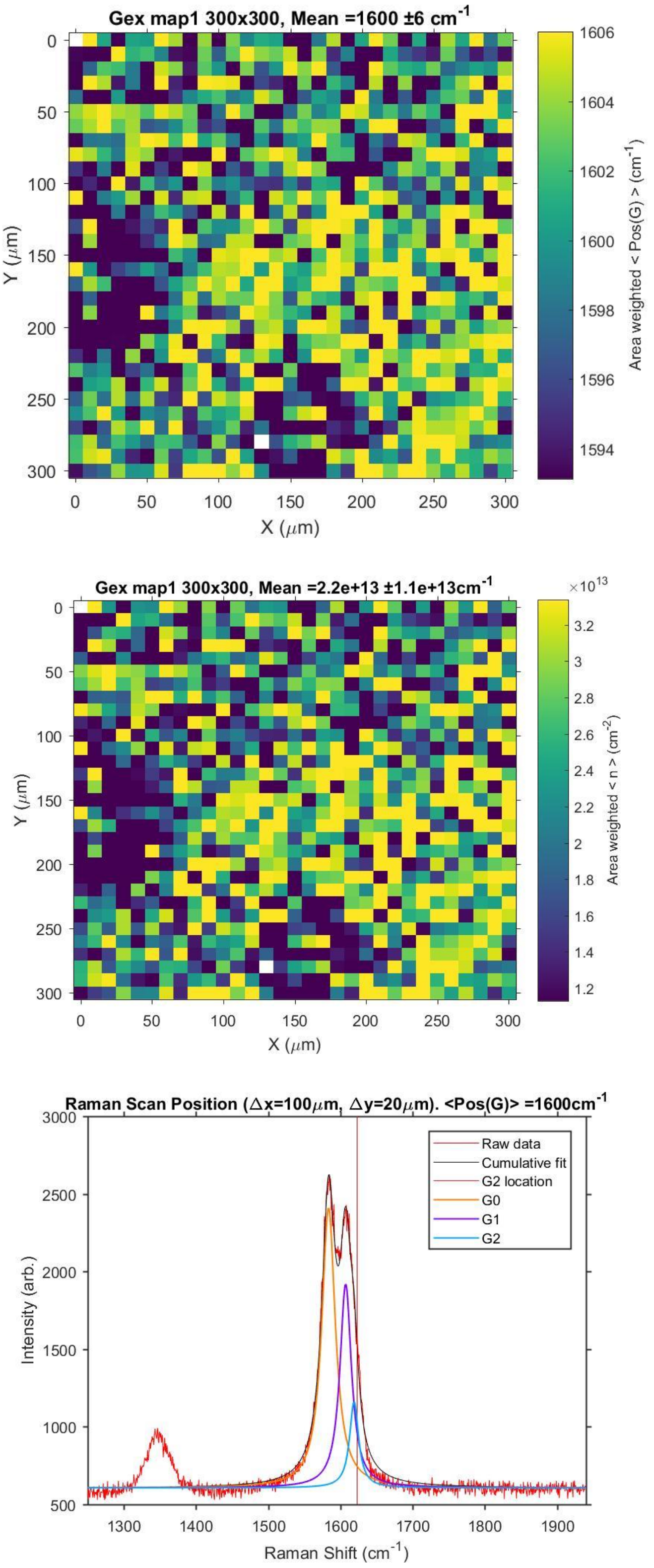

Supplementary Fig. S1. Extracting carrier density from Raman data. A) Raman map showing the extracted position of the area weighted mean G-peak for the intercalated few-layer graphene Sample B. Here, each data point corresponds to a full Raman spectrum. B) Extracted carrier density map, based on the extracted mean G-peak position, following Ref. **[S2]**. We find an average carrier density of $n = 2.2 \times 10^{13}\,\mathrm{cm}^{-2}$, corresponding to a Fermi energy of 0.55 eV. C) Example of a Raman spectrum with three G peaks around $1600\,\mathrm{cm}^{-1}$ identified by

Lorentzian functions. The splitting into multiple G peaks arises from the different stages of $FeCl_3$ intercalation, which leads to charge transfer and a doping-induce shift in G peak position **[S2]**.

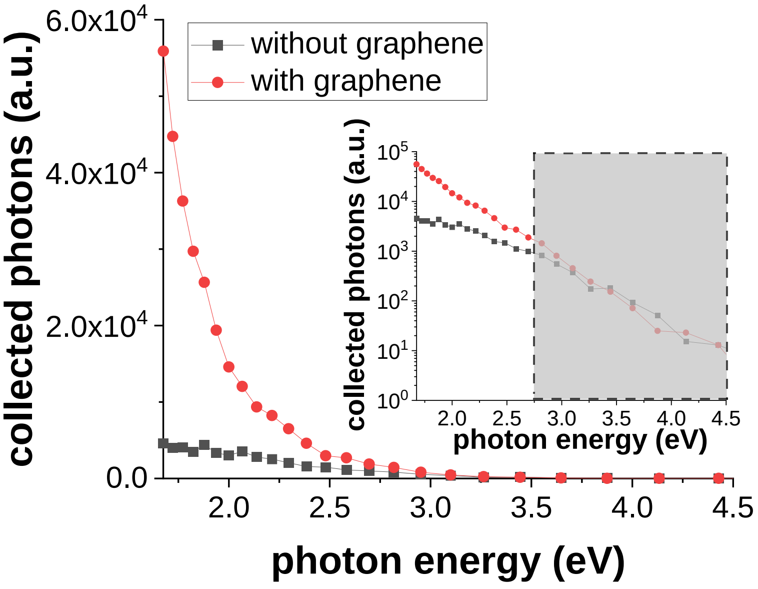

Supplementary Fig. S2. Optical emission from metamaterial grating samples containing a substrate covered by metallic grating with and without graphene.

Supplementary References

[S1] Bointon, T.; Jones, G.; De Sanctis, A.; Hill-Pearce, R.; Craciun M. F.; Russo, S. Large-area functionalized CVD graphene for work function matched transparent electrodes. *Sci. Rep*. **2015**, 5, 16464.

[S2] Khrapach, I.; Withers, F.; Bointon, T. H.; Polyushkin, D. K.; Barnes, W. L.; Russo, S.; Craciun, M. F. Novel Highly Conductive and Transparent Graphene-Based Conductors, *Adv. Mater*. **2012**, 24, 2844–2849.

[S3] Stepanov, A. G.; Kuhl, J.; Kozma, I. Z.; Riedle, E.; Almási, G.; and Hebling, J. Scaling up the energy of THz pulses created by optical rectification. *Opt. Express* **2005**, 13, 5762-5768

[S4] Deinert, J.-C.; Iranzo, D. A.; Pérez, R.; Jia, X.; Hafez, H. A.; Ilyakov, I.; Awari, N.; Chen, M.; Bawatna, M.; Ponomaryov, A. N.; Germanskiy, S.; Bonn, M.; Koppens, F. H. L.; Turchinovich, D.; Gensch, M.; Kovalev, S.; Tielrooij, K.-J. Grating-Graphene Metamaterial as a Platform for Terahertz Nonlinear Photonics, *ACS Nano* **2021**, 15, 1145−1154.

[S5] Massicotte, M.; Soavi, G.; Principi, A.; Tielrooij K.-J. Hot carriers in graphene – fundamentals and applications. *Nanoscale* **2021**, 13, 8376-8411.
